# Inflammatory Serum Proteins Are Severely Altered in Metastatic Gastric Adenocarcinoma Patients from the Chinese Population

**DOI:** 10.1371/journal.pone.0123985

**Published:** 2015-04-17

**Authors:** Jiangnan Wang, Rong Ma, Ashok Sharma, Mingfang He, Jing Xue, Jianzhong Wu, Boying Dun, Gang Li, Xiaoxiao Wang, Minghua Ji, Jin-Xiong She, Jinhai Tang

**Affiliations:** 1 Institute of Translational Medicine, School of Pharmaceutical Sciences, Nanjing University of Technology, Nanjing, Jiangsu Province, China; 2 Zhengjiang Jintai Biosciences, Zhengjiang, Jiangsu Province, China; 3 Jiangsu Cancer Hospital, Nanjing Medical University, Nanjing, Jiangsu Province, China; 4 Center for Biotechnology and Genomic Medicine, Medical College of Georgia, Georgia Regents University, Augusta, Georgia, United States of America; University of Hong Kong, HONG KONG

## Abstract

**Background:**

Inflammation is one of the major hallmarks of cancer. This study was designed to profile a panel of inflammatory mediators in gastric adenocarcinoma (GA) and to identify their potential differences separately in metastatic and non-metastatic patient subgroups.

**Methods:**

Serum samples from 216 GA patients and 333 healthy controls from China were analyzed for six proteins using the Luminex multiplex assay.

**Results:**

The serum levels for all the six proteins were significantly elevated in metastatic GA compared to non-metastatic GA. Two acute phase proteins (SAA and CRP) and a CXC chemokine (GRO) were significantly elevated in metastatic GA (p <0.01) but smaller changes were observed in non-metastatic GA compared to healthy controls. OPN is moderately increased in non-metastatic GA (2.05-fold) and more severely elevated in metastatic GA (3.34-fold). Surprisingly, soluble VCAM1 and AGP were significantly lower in both non-metastatic and metastatic GA patients compared to controls. Several individual proteins were shown to possess moderate diagnostic value for non-metastatic GA (AUC = 0.786, 0.833, 0.823 for OPN, sVCAM1 and AGP, respectively) and metastatic GA (AUC = 0.931, 0.720, 0.834 and 0.737 for OPN, sVCAM1, SAA and CRP, respectively). However, protein combinations further improve the diagnostic potential for both non-metastatic GA (best AUC = 0.946) and metastatic GA (best AUC = 0.963). The protein combination with best AUC value for both comparisons is OPN+sVCAM1+AGP+SAA.

**Conclusions:**

These results suggest that several serum proteins are directly related to the severity of gastric cancer. Overall, stronger associations are observed with metastatic than non-metastatic GA as the protein changes are greater with the metastatic status. A combination of these serum proteins may serve as non-invasive markers to assess the severity status and stage of gastric cancer.

## Introduction

Gastric cancer is one of the most common malignancies. It is ranked second as a cause of cancer mortality worldwide. More than 90% of the gastric cancers are adenocarcinomas [1]. China accounts for 42% of all gastric cancer cases of the world, about 300,000 Chinese dying from gastric cancer every year during the past decade [2, 3]. The prognosis is much worse for gastric cancer with distant metastasis than for localized gastric cancer. About one third of the gastric cancer patients are diagnosed with distant metastasis [4]; moreover, occult distant metastasis often escape detection by conventional techniques, which may be the main reason for the low survival rate. Early detection remains the most promising strategy to improve the survival of patients with gastric cancer. Studies investigating the impact of preoperative serum tumor markers for assessing patients with gastric cancer have shown that three markers (CEA, CA19-9 and CA72-4) are significantly associated with tumor stage and patient survival [5, 6]. However, these serum markers are not useful for early detection of cancer. Other serum markers associated with gastric cancer are CA50, STN, CA125, AFP, IAP, and TPA [7–10]. Many studies have demonstrated the clinical significance of each marker, however, low rates of sensitivity and specificity prevent the use of any of these serum in clinical setting. Identification of serum biomarkers is critical to the early diagnosis and the detection of metastasis in patients with gastric cancer.

Inflammation is one of the major hallmarks of cancer. Chronic inflammation increases the risk of malignancy. Tumor growth induces an inflammatory microenvironment and cancer cells can increase the production of inflammatory proteins [11, 12]. Thus, inflammation associated proteins may serve as potential biomarkers to predict aggressiveness and/or severity of various cancers. Identification of such biomarkers may help distinguish cancer patients with a greater risk of metastasis or disease recurrence. Serum amyloid A (SAA), C-reactive protein (CRP) and α1-acid glycoprotein (AGP) are all inflammatory markers that have been associated with malignant diseases. They are non-specific, acute-phase proteins secreted in response to cytokines such as IL-1, IL-6 and TNF-α [13–15]. Serum SAA and CRP levels are reported to be elevated in various cancers [16–21]. SAA has been suggested as an indicator of distant metastases but not as an early tumor marker in patients with renal cell carcinoma [22]. CRP was shown to be significantly higher only in patients with bone metastasis, but not with localized prostate cancer when compared with controls with benign prostatic hypertrophy [23]. These studies indicate that SAA and CRP may function as metastasis markers. AGP is a major serum glycoprotein with highly branched N-linked glycans. It has been demonstrated that the glycoforms of AGP changed during acute and chronic inflammation, pregnancy, estrogen treatment, and cancer [24]. Patients with advanced malignancies who had AGP glycoforms that contained highly fucosylated triantennary and tetraantennary sugar chains are likely to have a poor prognosis [25]. However, AGP expression and function in patients with malignancies remain undefined.

Osteopontin (OPN) is a multifunctional, calcium-binding glyco-phosphoprotein which participates in many biological processes such as inflammation, angiogenesis, tumor progression, and metastasis. The elevated serum/plasma OPN level has been found in a variety of human cancers [26–29]. Meanwhile, OPN is known as a metastasis regulator [30] and proposed to be a marker of bone metastasis in prostate cancer [31].

Within the tumor stroma, chemokines are thought to play an important role in the recruitment of immune cells to sites of inflammation. Growth-related oncogene (GRO), one of the ELR-positive subgroup of CXC chemokines, has been reported to be expressed by tumor tissues such as breast cancer, esophageal cancer, malignant melanoma and colon cancer [32–35]. The impact of GRO on tumor progression and clinical significance is still unclear to date.

In many disease processes, adhesion molecules facilitate the entry of leukocytes into inflamed tissues, which in turn promotes neovascularization, resulting in tumor growth and wound repair [36]. Vascular cell adhesion molecule-1 (VCAM1), a member of the immunoglobulin superfamily of adhesion molecules which is up-regulated in response to tumor necrosis factor-alpha, IL-1 and lipopolysaccharide, plays key roles in various stages of tumor angiogenesis and is also involved in tumor progression and metastasis [37, 38].

Although several of these inflammatory proteins have been studied in various types of cancer, their status and potential role in GA are not yet well understood. The goal of the current study was to assess the serum levels of a panel of inflammatory markers (SAA, CRP, AGP, GRO, OPN and sVCAM1) in a large number of GA patients from a Chinese population, with a special emphasis on the potential difference between metastatic and non-metastatic patients.

## Methods

### Human subjects and serum samples

A total of 216 newly-diagnosed patients with gastric adenocarcinoma (ages 59.80 ± 10.43 years) prior to any therapy, and 333 healthy control subjects (ages 50.15 ± 10.3 years) were involved in the present study. Malignant tumors were staged according to the American Joint Committee on Cancer (AJCC) TNM classification (7th version, 2010). The characteristics of the patients are summarized in Table 1. Blood samples of gastric cancer patients were obtained from Jiangsu Cancer Hospital prior to the initiation of any treatment. Samples were centrifuged for 10min at 3,000 rpm at 4°C, and serum was subsequently frozen at -80°C until use. This study has been approved by the human subject ethics committee of the Jiangsu Cancer Hospital and informed consent signed by the study subjects.

**Table 1:** Characteristics of the patient population.

		Healthy Controls (n = 333)	GC: All (n = 217)	GC: NM (n = 103)	GC: M (n = 64)
Age(year)	Mean ± SD	50.15 ± 10.3	59.80 ± 10.43	57.90 ± 9.87	59.60 ± 11.60
	Median (Range)	49 (35–80)	61 (25–81)	59 (31–80)	61 (25–81)
FIGO staging	Stage I+II		37	37	0
	Stage III		65	65	0
	Stage IV		64	0	64
	Undetermined		51	1	0
Gender	Female		73	36	24
	Male		143	66	40
Differentiation	Poor		72	38	23
	Moderate		78	59	14
	High		5	0	0
	Undetermined		62	6	27
Tumor Region	Cardia		77	30	18
	Gastric Body		54	28	20
	Gastric Antrum		40	25	9
	Stomach Angle		21	16	0
	Other		25	4	17
Morphology	Ulcerative type		78	61	11
	Infiltrating type		22	18	4
	Uplift type		11	0	5
	Other		106	24	44
Vascular Invasion	Yes		141	80	61
	No		25	22	3
	Undetermined		51	1	0
Nerve Invasion	Yes		123	59	63
	No		44	43	1
	Undetermined		50	1	0
Lymph node metastasis	Yes		111	64	37
	No		65	37	27
	Undetermined		41	2	0

### Luminex assays

Luminex assays for OPN, AGP, SAA, CRP, GRO and sVCAM1 were obtained from Millipore (Millipore Inc, Billerica, MA, USA). The assays were performed according to the manufacturer’s instructions. Briefly, serum samples were incubated with antibody-coated microspheres, followed by biotinylated detection antibody. Proteins were detected by incubation with phycoerythrin-labeled streptavidin and the resultant bead immuno-complexes were read on a FLEXMAP3D (Luminex, TX, USA) with the following instrument settings: events/bead: 50, minimum events: 0, Flow rate: 60ul/min, Sample size: 50ul, discriminator gate: 8000–13500. Median fluorescence intensity (MFI) was collected and used for calculating protein concentration.

### Statistical analysis

Protein concentrations were estimated using a regression fit to the standard curve with known concentration included on each plate using a serial dilution series. The concentrations were logarithmically transformed prior to all statistical analyses, to achieve normal distribution. The differences between two groups were examined using an unpaired t-test or Mann-Whitney test. The comparisons for ≥3 groups were made by ANOVA followed by pair-wise comparisons using Bonferroni post hoc testing. The statistical significance of differences was set at P<0.05. To examine the relationships between disease status and the serum protein levels, logistic regression was used by including age and sex as co-variates. The pairwise correlations were computed using Pearson correlation. Clustering and visualization of correlation matrix was performed using hierarchical clustering method and heatmap. The diagnostic power of individual proteins and their combinations was assessed using the receiver operating characteristic (ROC) curve and area under the curve (AUC) was calculated. To assess the odds ratios of having GA at different levels of each protein, subjects were divided into five quintiles based on protein levels. The cutoff protein levels for these quintiles were then used to count controls and cases in each quintile. The 1st quintile was used as reference and odds ratios of having disease was calculated for upper four quintiles using Pearson's chi-squared test with Yates' continuity correction. The chi-squared test for trend in proportions was used to calculate the p-value of overall trend. Risk scores (equal to odds ratio) were assigned to each subject based on individual protein levels. For a combination of proteins, the combined risk score of each subject was calculated by simply adding risk score from multiple proteins. The odds ratios of having disease were calculated for upper four quintiles against the bottom quintile as mentioned above. All statistical analyses were performed using the R language and environment for statistical computing (R version 2.15.1; R Foundation for Statistical Computing; www.r-project.org).

## Results

### Serum protein changes in gastric adenocarcinoma

**Fig 1:**
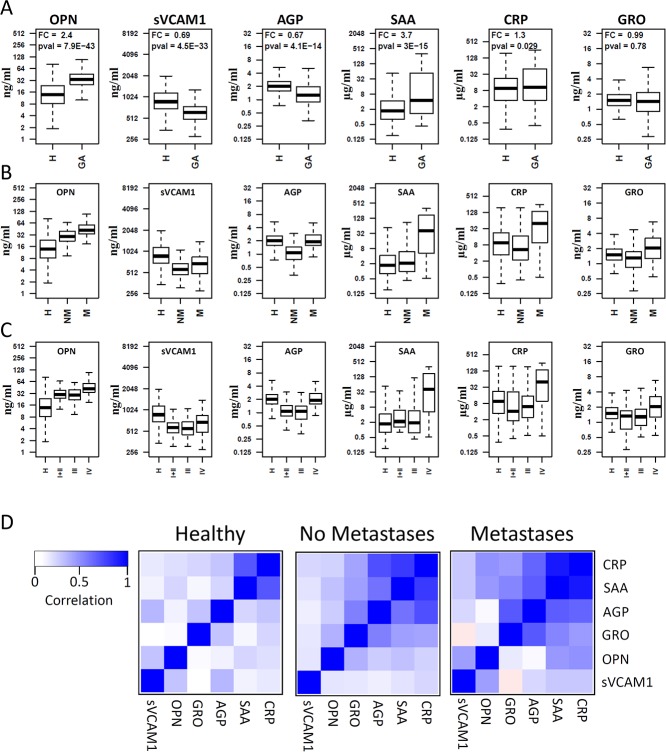
Boxplots representing the serum protein levels in patient subgroups and healthy controls. Subgroups are defined based on the presence of cancer (A), metastasis status (B), and cancer stage (C). H: Healthy controls, GA: Gastric Adenocarcinoma, NM: patients with no metastasis, M: patients with distant metastasis. The pairwise correlations between all six proteins are shown in H, NM, and M subgroups (D). Correlations between individual protein levels were computed using Pearson correlation coefficient. Clustering and visualization of correlation matrix was performed using hierarchical clustering method and heatmap. Overall strong positive correlations were observed in both the GA patients with distant metastasis and without distant metastasis as compared to healthy controls.

Six candidate proteins (OPN, sVCAM1, SAA, CRP, AGP and GRO) were measured in serum samples from 219 newly-diagnosed patients with gastric adenocarcinoma and 333 healthy control subjects using Luminex multiplex assays. The raw data are presented as box plots in Fig 1A. Three proteins (OPN, SAA and CRP) were significantly increased in GA patients compared to healthy controls. The mean OPN level is 2.4-fold higher in GA patients than controls (p<10^-42^). The mean SAA level is 3.7-fold higher in GA patients than controls (p<10^-14^), while the increase in mean CRP level is barely significant (FC = 1.3, p = 0.029). sVCAM1 is lower in GA patients than controls (FC = 0.69, p<10^-32^). Furthermore, the mean level of AGP is also lower in GA patients (FC = 0.67, p<10^-13^). No significant difference was revealed for GRO between GA patients and controls.

Logistic regression was carried out using protein concentration as dependent variable and sex and age as covariates. As shown in Table 2, four of the five proteins remain significantly associated with GA even after adjusting for age and sex with the exception of CRP which was barely significant before adjustment. These results suggest that the association between GA and these serum proteins are not due to the covariates examined in this study.

**Table 2:** Logistic Regression Analysis using serum protein concentration, before and after adjusting for covariates.

	Unadjusted			Adjusted for age and gender		
Protein	OR	95% CI	p-value	OR	95% CI	p-value
OPN	4.275	(3.266–5.597)	4.09 x 10^-26^	3.988	(2.998–5.304)	2.04 x 10^-21^
sVCAM1	0.110	(0.071–0.171)	8.19 x 10^-23^	0.042	(0.023–0.077)	3.81 x 10^-25^
AGP	0.364	(0.275–0.483)	1.97 x 10^-12^	0.287	(0.198–0.414)	2.76 x 10^-11^
SAA	1.364	(1.262–1.474)	4.48 x 10^-15^	1.356	(1.246–1.476)	1.76 x 10^-12^
CRP	1.093	(1.006–1.187)	0.035	1.047	(0.956–1.146)	0.321
GRO	0.964	(0.741–1.255)	0.787	0.770	(0.487–1.215)	0.261

### Correlations between serum proteins and clinico-pathological characteristics

We examined associations between protein levels and clinico-pathological characteristics including tumor size, tumor invasion depth, TNM staging, differentiation degree, lymph node metastasis, vascular invasion, nerve invasion and distant metastasis. Among these characteristics, distant metastasis and stage have a major impact on serum protein levels. As shown in Fig 1B, significant differences were observed between GA patients with distant metastasis (M) and no distant metastasis (NM). The serum levels for all the six proteins were significantly elevated in metastatic patients compared to non-metastatic patients. The most striking difference is observed for AGP which is significantly higher in metastatic patients than non-metastatic patients (FC = 1.78, p<10^-8^). SAA is also highly elevated in patients with metastasis compared to both healthy controls (FC = 12.4, p<10^-13^) and patients without metastasis (FC = 6.98, p<10^-8^) (Fig 1B). Similarly, CRP is significantly increased only in patients with metastasis (FC = 3.6, p<10^-7^) compared to healthy controls.

As expected, stage IV patients, which are defined by the presence of distant metastasis, have significantly higher serum levels for all six proteins compared to the earlier stages (Fig 1C). However, no significant difference was observed for any of the six proteins between stage I, II and III (Fig 1C), suggesting that metastasis is accompanied by dramatic changes in serum proteins.

Because the serum protein concentrations were distinctly elevated in gastric cancer patients with distant metastasis compared to patients without distant metastasis, we separated the subjects into three groups: healthy controls (H), GA patients without distant metastasis (NM) and GA patients with distant metastasis (M). Correlations were calculated between each pair of the six proteins for these three groups separately. The correlation matrix was then subjected to hierarchical clustering to identify the clusters of correlated proteins (Fig 1D). Our data indicate much higher correlations among these proteins in both the GA patients with distant metastasis and without distant metastasis.

### Influence of different protein levels on GA risk

**Fig 2:**
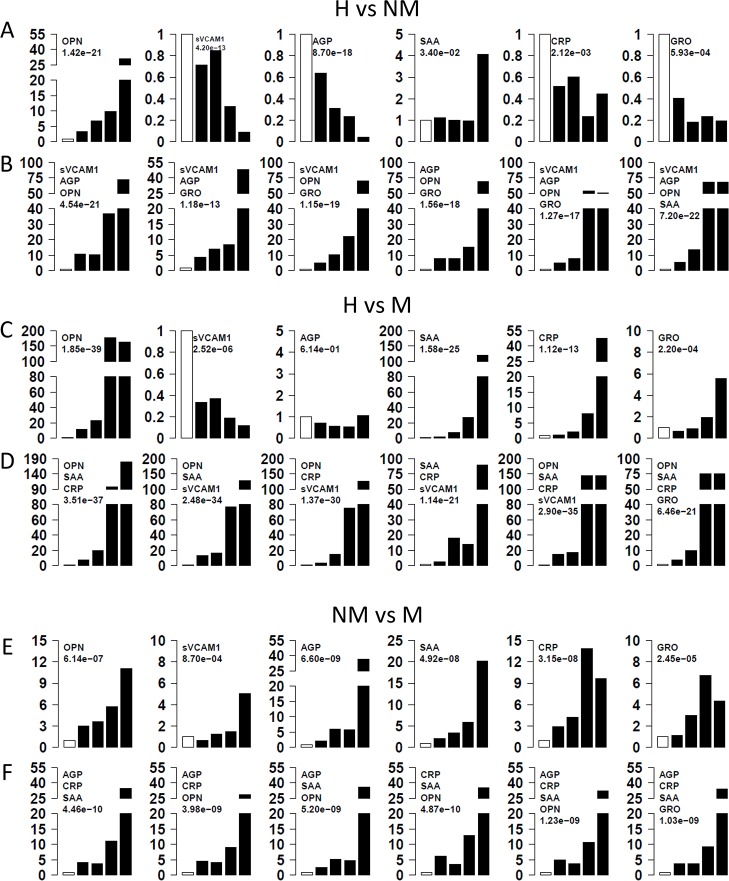
Risk of gastric adenocarcinoma and metastasis with protein alterations. H: Healthy controls, NM: patients with no metastasis, M: patients with distant metastasis. Three comparisons were made separately: H *vs* NM (A, B), H *vs* M (C, D), and NM *vs* M (E, F). Subjects were divided into five quintiles based on individual protein levels. The 1st quintile was used as reference and odds ratios of having disease was calculated for upper four quintiles (A, C, E). The chi-squared test for trend in proportions was used to calculate the p-value of overall trend. For a combination of proteins (B, D, F) the combined risk score of each subject was calculated by simply adding risk score from multiple proteins and odds ratios were computed.

To assess the relationship between GA and different levels of serum proteins, we calculated the odds ratios associated with different levels of serum proteins. Firstly, we divided GA patients without distant metastasis into five quintiles and determined the quintile cutoff values, which were then used to assign the controls into the corresponding quintiles. The bottom quintile was used as reference and compared to each of the other four quintiles. The odds ratios for each protein are charted in Fig 2A. The strongest association is observed with OPN (p-trend<10^-20^). The odds ratio for GA increases with increasing OPN levels, reaching an OR of ~30 for the 5^th^ quintile. SAA is associated with GA without distant metastasis only in the 5^th^ quintile (OR = 4, p-trend = 0.034). Interestingly, increasing levels of sVCAM1, AGP and GRO levels are associated with reduced risk for GA without metastasis.

Similar analyses were conducted for GA patients with distant metastasis. For these analyses, quintile cutoff values were derived from GA patients with metastasis and applied to healthy control subjects. The bottom quintile was used as reference to calculate odds ratios for metastatic GA patients (Fig 2C). Overall, stronger associations are observed with metastatic than non-metastatic GA for all proteins except AGP. The odds ratios for OPN reached 170 for the 4^th^ and 5^th^ quintile (p-trend<10^-38^) for metastatic GA. Odds ratio also reached 120 for SAA for the 5^th^ quintile and 27 for the 4^th^ quintile (p-trend<10^-24^), while odds ratios reached 47 for the 5^th^ quintile and 8 for the 4^th^ quintile for CRP. In contrast to the observation in non-metastatic GA, increasing GRO level is associated with increasing risk for metastatic GA (OR = 6 for the 5^th^ quintile, p = 0.00022). Similar to non-metastatic GA, increasing sVCAM1 is associated with decreasing risk for metastatic GA.

Finally, we evaluated the potential differences between metastatic and non-metastatic GA using quintile cutoffs for metastatic GA. As shown in Fig 2E, higher protein levels are associated with increased odds ratio for metastatic GA for all six proteins. The largest differences are observed with AGP, SAA and then CRP and OPN.

### Effect of protein combinations on GA risk

Since multiple serum proteins are associated with GA, we attempted to examine the combined effect of these proteins on GA. Odds ratios calculated in previous step using quintiles were used as risk score for each subject based on the protein level. Combined risk score was calculated for each subject by adding the quintile odds ratios for multiple proteins. The associations between GA and the combined risk scores were investigated accordingly. We examined the risk scores based on three-protein and four-protein combinations. In “H vs NM” group (Fig 2B), multiple models improved the risk stratification. For example, one combination (OPN-sVCAM1-AGP-SAA) improve the highest OR values to 70 for both the 4^th^ and 5^th^ quintile (p<10^-21^) (Fig 2B).

In the “H vs M” comparison (Fig 2D), several models performed well but none of the models out-performed the single protein OPN, suggesting that OPN is a very important protein that can distinguish metastatic GA patients from healthy controls (Fig 2D). In “NM vs M” comparison (Fig 2F), the three-protein or four-protein combinations slightly improved the performance over single proteins.

### Diagnostic value of serum proteins

**Fig 3:**
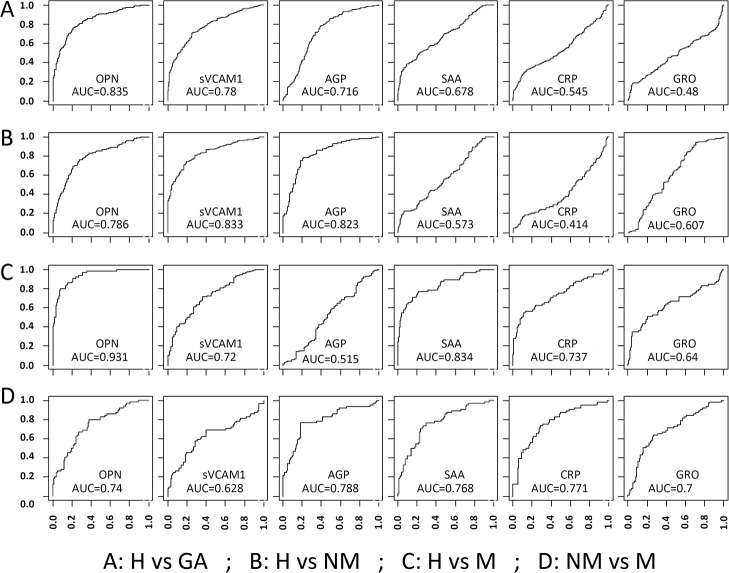
ROC Curves to evaluate the utility of six candidate biomarkers for GA and associated distant metastasis. H: Healthy controls, GA: Gastric Adenocarcinoma, NM: patients with no metastasis, M: patients with distant metastasis. Four comparisons were made separately: H *vs* GA (A), H *vs* NM (B), H *vs* M (C) and NM vs M (D). For comparisons with non-metastasis and healthy controls, the best AUC values are 0.833 (sVACM1) and 0.786 (OPN). For comparisons with metastasis and healthy controls, the best AUC values are 0.931 (OPN) and 0.834 (SAA).

The potential utility of serum proteins as GA biomarkers was evaluated using the areas-under-curve (AUC) in receiver-operating-characteristic (ROC) curves. We first evaluated each of the six proteins for their ability to distinguish: 1) all GA patients from controls (H vs GA), 2) non-metastatic patients from healthy controls (H vs NM), 3) metastatic patients from healthy controls (H vs M), and 4) metastatic from non-metastatic patients (NM vs M). As shown in Fig 3, individual proteins generally do not possess great diagnostic value with a few exceptions. The best AUC values are 0.835 (OPN) in the H vs GA comparison, 0.833 (sVCAM1) and 0.823 (AGP) in the H vs NM comparison, 0.931 (OPN) and 0.834 (SAA) in the H vs M comparison, and 0.788 (AGP) and 0.771 (CRP) in the NM vs M comparison.

**Fig 4:**
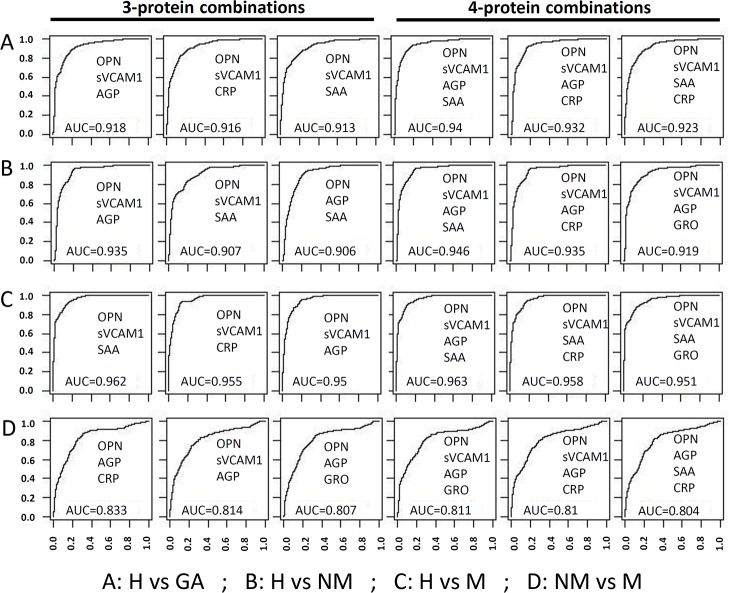
The utility of protein combinations to distinguish GA and associated distant metastasis. For multi-protein models, linear discriminate analysis was performed using 3 protein and 4 protein combinations. The diagnostic performance of each model was evaluated using leave one out cross validation method. The utility of serum proteins as cancer biomarkers was evaluated using the area-under-curve (AUC) of the ROC curves for different models. Several protein combinations possess excellent diagnostic value (AUC >0.9) in distinguishing both non-metastatic and metastatic samples from healthy controls.

Subsequently, we attempted to improve the AUC values using combinations of proteins. Combined risk score was calculated for each subject by adding the quintile odds ratios for multiple proteins as described above. The potential diagnostic value of combined risk score was evaluated using the ROC curves. As shown in Fig 4, multiple combinations can improve the AUC values for all four comparisons. For the H vs NM comparison the three protein model with best AUC of 0.935 is OPN-sVCAM1-AGP and the four protein model with best AUC of 0.946 is OPN-sVCAM1-AGP-SAA. For the H vs M comparison the three protein model with best AUC of 0.962 is OPN-sVCAM1-SAA and the four protein model with best AUC of 0.963 is OPN-sVCAM1- SAA-AGP model. However, the best models for NM vs M comparison only had AUC around 0.833.

S1–S4 Tables present the sensitivity values for different proteins and models at four different specificity thresholds (90%, 95%, 99% and 100%). For non-metastatic GA, sVCAM1 has the best performance, reaching a sensitivity of 58.9% and 50.7% at 90% and 95% specificity, while OPN is the next best performing molecule (S2 Table). However, the multivariate models only slightly improved the sensitivity, achieving a best sensitivity of 82.6% and 73% at 90% and 95% specificity. For metastatic GA, the best performing molecule is OPN, achieving a sensitivity of 79.7% and 67.2% at 90% and 95% specificity, respectively (S3 Table). However, the best performing model significantly improved the sensitivity to 88.1% and 76.8% at 90% and 95% specificity, respectively. The best molecule to distinguish metastatic from non-metastatic GA is AGP, achieving sensitivity values of 42.2% and 34.4% at specificity thresholds of 90% and 95%, respectively (S4 Table). The multivariate models also improved the sensitivity, achieving a best sensitivity of 53.1% and 42.5% at 90% and 95% specificity.

## Discussion

GA is a major malignant disease with high mortality in China [2, 3]. It is associated with a high incidence of metastasis which contributes to the low survival rate [4]. Identification of metastatic patients is important for therapeutic decisions. A total of nine serum markers (CEA, CA19-9, CA72-4, CA50, STN, CA125, AFP, IAP, and TPA) are known for gastric cancer monitoring [5–10]. Literature survey suggests that currently, a combinations of CEA, CA19-9, and CA72-4 are the most useful for managing gastric cancer [39]. CA 72–4 showed a higher positivity rate for gastric cancer (47.7%) than CEA (25%), and CA 19–9 (25%). The combination of CA 72–4 with CEA and CA 19–9 increased the sensitivity to 61.4% [40]. These tumor markers continue to have only limited diagnostic usefulness in monitoring gastric cancer patients mainly due to low sensitivity and specificity. In this study we identified six new markers which have not been used previously for monitoring gastric cancer patients. For non-metastatic GA, three markers with best AUC are OPN (0.786), sVCAM1 (0.833) and AGP (0.823). For metastatic GA, three markers with best AUC are OPN (0.931), SAA (0.834) and CRP (0.737). Overall, OPN has excellent diagnostic value for monitoring gastric cancer for both non-metastatic and metastatic GA. The protein combination OPN +sVCAM1 +AGP +SAA has the best AUC value for both non-metastatic (0.946) and metastatic GA (0.963).

Inflammatory microenvironment exists in all tumors and inflammatory response plays decisive roles at different stages of tumor development including initiation, promotion, malignant conversion, invasion, and metastasis [12]. Inflammation-associated proteins such as cytokines, chemokines [11] and acute-phase proteins [41] have been found to be increased in malignancies. *Helicobacter pylori* infection is the main risk factor for gastric cancers and the mechanism by which *H*. *pylori* induces stomach cancer potentially involves chronic inflammation. CRP and SAA are two very important acute-phase proteins whose concentrations increase in response to inflammation [42, 43]. These proteins, especially CRP, have been extensively investigated in various types of cancer. CRP was reported to be higher in metastatic compared to localized prostate cancer [23]. Only one previous study with a very small number of cases (n = 17) suggested higher SAA and CRP levels in patients with metastatic gastric cancer than in those with limited disease [41]. The current study with 219 GA patients and 333 healthy controls convincingly demonstrates that CRP is significantly elevated in metastatic GA but not in non-metastatic GA when compared to healthy controls. This is the first report of drastic elevation of SAA in metastatic GA (12-fold) but moderate elevation in non-metastatic GA (1.8-fold). ROC analysis suggested SAA as a good marker to separate metastatic from non-metastatic tumors (AUC = 0.768). Similarly, CRP can be used to separate metastatic from non-metastatic tumors (AUC = 0.771). This study also demonstrates a high positive correlation between SAA and CRP concentrations, with stronger correlations in the patient group than in controls. The coordinated elevation of SAA and CRP in patients could be explained by the inflammatory response to tumor. Moreover, tumor cells have also been found to express CRP and SAA [44–46], which may explain the higher correlation between SAA and CRP in metastatic tumors than in non-metastatic tumors.

AGP, another acute phase protein, is a heavily glycosylated lipocalin. Its serum concentration increases in response to systemic tissue injury, inflammation or infection. AGP is synthesized by the liver as well as other cells such as endothelial cells and leukocytes [47, 48]. The biological function of AGP is poorly understood. Differential glycosylation may be a prognostic biomarker for cancer [25]. In our study, serum AGP is significantly higher in metastatic patients than non-metastatic patients, suggesting that AGP may be used as a potential biomarker for distant metastasis (AUC = 0.788). However, it was surprising that AGP levels were actually lower in non-metastatic patients compared to healthy controls. Therefore, the role of AGP in GA still needs to be further investigated.

GRO, a member of the CXC chemokine family, was originally identified as a growth stimulating regulator [49]. Previous reports have suggested the involvement of the *GRO* gene in tumor growth and metastasis of colon cancer and squamous cell carcinoma [50, 51]. Non-metastatic and low metastatic cells express lower levels of GRO as compared to high metastatic colon carcinoma cells [51]. This is the first report of GRO in GA and our observation of higher GRO in serum samples of metastatic GA patients is consistent with the previous findings on GRO gene expression in tumor tissues. Our results suggest that GRO may be a useful biomarker for metastatic GA.

The activity and expression of VCAM1 is reported to be up-regulated following inflammation. sVCAM1 was significantly elevated in patients with Stage 4 breast cancer compared with controls, whereas the origin of soluble adhesion molecules is unclear [38]. In our study, serum sVCAM1 is significantly higher in metastatic patients than non-metastatic patients. However, sVCAM1levels were lower in non-metastatic patients compared to healthy controls. The role of sVCAM1 in GA and the origin of sVCAM1 need further investigation.

Among the proteins analyzed in this study, OPN showed the strongest association with GA. The mean OPN level in all GA patients is 2.4-fold higher in patients than in healthy controls. OPN levels are also significantly higher in metastatic patients than non-metastatic patients. Increasing OPN levels are associated with increasing risk for both non-metastatic and metastatic GA. OPN has an AUC value of 0.786 to separate non-metastatic GA patients from controls and an AUC of 0.931 to separate metastatic cancers from controls, suggesting that OPN has excellent diagnostic value for both types of GA. Furthermore, OPN also has an AUC of 0.74 to separate metastatic and non-metastatic cancers. Our findings are consistent with previous reports indicating that high OPN levels are significantly associated with metastasis in different types of cancers [30]. Although there are numerous reports indicating the implication of OPN in GA, there was only one study suggesting that serum/plasma OPN is significantly increased in GA and that high level of OPN is associated with poor prognosis [52]. This study thus provides strong support for OPN as a GA serum biomarker that should be further explored for its clinical use. OPN has a variety of biological functions including bone remodeling, chemotaxis, cell activation and apoptosis. OPN is reported to act as an immune modulator in a variety of manners such as promotion of immune cell recruitment to inflammatory sites, inhibition of Th2 cytokine production and enhancement of Th1 cytokine production.

It is well known that using multiple molecules (or models) may significantly improve the performance of biomarkers. In this study, we evaluated the performance of several three-protein and four-protein combinations. Several models had AUC value of >0.9 for non-metastatic GA (stage I, II, III) versus healthy controls. As stage I-II patients are not different from stage III patients, these data reflect the performance for early stage GA. Therefore, these biomarkers are potentially useful for early disease detection. The highest AUC was 0.963 in the metastatic GA versus healthy controls, suggesting that the biomarkers perform better for later stage cancer.

In summary, this study identified six inflammatory proteins that are significantly altered in the serum of early stage GA patients and more severely altered in the late stage GA patients. Several combinations of these proteins may be useful biomarkers for GA.

## Supporting Information

Area under the curve (AUC) and sensitivity of individual proteins and combinations of proteins

S1 Table Healthy controls and all GA samples (H *vs* GA). (PDF)

S2 Table Healthy controls and non-metastatic GA samples (H *vs* NM). (PDF)

S3 Table Healthy controls and metastatic GA samples (H *vs* M). (PDF)

S4 Table Non-metastatic and metastatic samples (NM *vs* M). (PDF)
