# Supplementary material for: Inflammatory Serum Proteins Are Severely Altered in Metastatic Gastric Adenocarcinoma Patients from the Chinese Population
Source: PLoS One. 2015 Apr 17;10(4):e0123985. doi: 10.1371/journal.pone.0123985 (PMC4401731; doi:10.1371/journal.pone.0123985)
Supplement: S1 Table — (PDF) [file pone.0123985.s001.pdf]

S1 Table: Area under the curve (AUC) and sensitivity of individual proteins and combinations of proteins between healthy controls and all GA samples (H vs GA).

Protein	AUC (95% CI)	p val	Specificity (%)			
			90	95	99	100
OPN	0.84 (0.82 - 0.85)	6.99 $x10^{-40}$	55.30	42.86	25.81	2.76
sVCAM1	0.78 (0.76 - 0.80)	1.59 $x10^{-28}$	49.24	39.88	19.64	10.57
AGP	0.72 (0.69 - 0.74)	1.43 $x10^{-15}$	19.34	13.99	3.29	1.23
SAA	0.68 (0.66 - 0.70)	1.94 $x10^{-12}$	38.43	31.94	13.43	7.41
CRP	0.55 (0.52 - 0.57)	7.42 $x10^{-2}$	27.65	17.51	11.52	3.23
GRO	0.48 (0.45 - 0.51)	4.97 $x10^{-1}$	19.07	18.60	4.19	0.93
**OPN+sVCAM1+AGP**	**0.92 (0.91 - 0.92)**	**$<10^{-99}$**	**73.55**	**61.87**	**17.43**	**1.14**
**OPN+sVCAM1+SAA**	**0.91 (0.91 - 0.92)**	**$<10^{-99}$**	**73.07**	**62.85**	**30.05**	**13.72**
**OPN+sVCAM1+CRP**	**0.92 (0.91 - 0.92)**	**$<10^{-99}$**	**73.25**	**60.36**	**20.81**	**8.98**
OPN+sVCAM1+GRO	0.90 (0.90 - 0.90)	$<10^{-99}$	70.82	61.94	10.92	3.07
OPN+AGP+SAA	0.90 (0.90 - 0.90)	$<10^{-99}$	67.10	47.34	9.63	0.00
OPN+AGP+CRP	0.88 (0.88 - 0.88)	$<10^{-99}$	62.92	48.33	8.85	0.00
OPN+AGP+GRO	0.85 (0.84 - 0.86)	$<10^{-99}$	56.26	37.76	10.29	0.00
OPN+SAA+CRP	0.84 (0.84 - 0.85)	$<10^{-99}$	53.70	41.86	25.72	3.11
OPN+SAA+GRO	0.83 (0.82 - 0.83)	$<10^{-99}$	55.84	36.83	23.27	15.67
OPN+CRP+GRO	0.82 (0.81 - 0.83)	$<10^{-99}$	51.66	38.32	18.69	8.02
**OPN+sVCAM1+AGP+SAA**	**0.94 (0.94 - 0.94)**	**$<10^{-99}$**	**82.47**	**69.06**	**34.63**	**0.00**
**OPN+sVCAM1+AGP+CRP**	**0.93 (0.93 - 0.94)**	**$<10^{-99}$**	**76.97**	**67.50**	**12.72**	**0.00**
OPN+sVCAM1+AGP+GRO	0.90 (0.89 - 0.90)	$<10^{-99}$	67.30	56.44	7.87	0.28
**OPN+sVCAM1+SAA+CRP**	**0.92 (0.92 - 0.93)**	**$<10^{-99}$**	**74.91**	**62.90**	**27.89**	**14.01**
OPN+sVCAM1+SAA+GRO	0.91 (0.91 - 0.92)	$<10^{-99}$	75.57	62.84	33.16	11.42
OPN+sVCAM1+CRP+GRO	0.89 (0.89 - 0.90)	$<10^{-99}$	70.54	56.86	34.83	8.54
OPN+AGP+SAA+CRP	0.91 (0.90 - 0.91)	$<10^{-99}$	71.40	47.20	13.87	0.00
OPN+AGP+SAA+GRO	0.89 (0.88 - 0.89)	$<10^{-99}$	59.83	41.92	9.66	0.00
OPN+AGP+CRP+GRO	0.86 (0.85 - 0.86)	$<10^{-99}$	57.05	47.92	6.20	0.00
OPN+SAA+CRP+GRO	0.84 (0.84 - 0.85)	$<10^{-99}$	58.07	38.06	20.24	7.37
