# Supplementary material for: Inflammatory Serum Proteins Are Severely Altered in Metastatic Gastric Adenocarcinoma Patients from the Chinese Population
Source: PLoS One. 2015 Apr 17;10(4):e0123985. doi: 10.1371/journal.pone.0123985 (PMC4401731; doi:10.1371/journal.pone.0123985)
Supplement: S2 Table — (PDF) [file pone.0123985.s002.pdf]

S2 Table: Area under the curve (AUC) and sensitivity of individual proteins and combinations of proteins between healthy controls and non-metastatic GA samples (H vs NM).

Protein	AUC (95% CI)	p val	Specificity (%)			
			90	95	99	100
OPN	0.79 (0.76 - 0.81)	2.17 $x10^{-18}$	42.72	30.10	15.53	0.00
sVCAM1	0.83 (0.81 - 0.85)	1.75 $x10^{-24}$	58.91	50.76	35.65	33.23
AGP	0.82 (0.80 - 0.84)	2.79 $x10^{-21}$	46.50	27.16	17.70	17.28
SAA	0.57 (0.54 - 0.60)	2.58 $x10^{-2}$	22.55	19.61	5.88	1.96
CRP	0.41 (0.38 - 0.44)	8.31 $x10^{-3}$	16.50	6.80	4.85	0.00
GRO	0.61 (0.57 - 0.64)	3.01 $x10^{-3}$	3.45	1.72	0.57	0.00
**OPN+sVCAM1+AGP**	**0.94 (0.93 - 0.94)**	**$<10^{-99}$**	**78.72**	**65.23**	**9.09**	**0.10**
**OPN+sVCAM1+SAA**	**0.91 (0.90 - 0.91)**	**$<10^{-99}$**	**71.32**	**63.73**	**24.65**	**3.56**
OPN+sVCAM1+CRP	0.90 (0.90 - 0.91)	$<10^{-99}$	71.37	56.30	16.87	2.94
OPN+sVCAM1+GRO	0.89 (0.89 - 0.90)	$<10^{-99}$	69.96	55.23	7.75	2.42
**OPN+AGP+SAA**	**0.91 (0.90 - 0.91)**	**$<10^{-99}$**	**66.31**	**46.29**	**3.52**	**0.00**
OPN+AGP+CRP	0.88 (0.87 - 0.89)	$<10^{-99}$	57.02	41.44	5.58	0.00
OPN+AGP+GRO	0.84 (0.84 - 0.85)	$<10^{-99}$	45.86	30.77	3.41	0.00
OPN+SAA+CRP	0.79 (0.78 - 0.80)	$<10^{-99}$	41.38	30.17	5.84	0.57
OPN+SAA+GRO	0.80 (0.79 - 0.80)	$<10^{-99}$	45.90	23.46	6.56	1.43
OPN+CRP+GRO	0.79 (0.78 - 0.80)	$<10^{-99}$	47.12	29.93	5.19	0.28
**OPN+sVCAM1+AGP+SAA**	**0.95 (0.94 - 0.95)**	**$<10^{-99}$**	**82.61**	**72.99**	**24.36**	**0.00**
**OPN+sVCAM1+AGP+CRP**	**0.94 (0.93 - 0.94)**	**$<10^{-99}$**	**79.65**	**64.59**	**12.67**	**0.00**
**OPN+sVCAM1+AGP+GRO**	**0.92 (0.91 - 0.92)**	**$<10^{-99}$**	**76.63**	**59.70**	**11.19**	**0.00**
OPN+sVCAM1+SAA+CRP	0.91 (0.90 - 0.91)	$<10^{-99}$	70.71	59.17	29.49	6.01
OPN+sVCAM1+SAA+GRO	0.90 (0.89 - 0.90)	$<10^{-99}$	70.10	54.70	14.70	6.10
OPN+sVCAM1+CRP+GRO	0.89 (0.89 - 0.90)	$<10^{-99}$	69.99	57.23	10.37	2.89
OPN+AGP+SAA+CRP	0.90 (0.89 - 0.91)	$<10^{-99}$	68.20	44.80	9.10	0.00
OPN+AGP+SAA+GRO	0.89 (0.88 - 0.89)	$<10^{-99}$	55.44	42.36	2.00	0.00
OPN+AGP+CRP+GRO	0.84 (0.83 - 0.85)	$<10^{-99}$	48.56	34.23	2.79	0.00
OPN+SAA+CRP+GRO	0.79 (0.78 - 0.80)	$<10^{-99}$	45.02	23.26	8.76	1.31
