# Supplementary material for: Inflammatory Serum Proteins Are Severely Altered in Metastatic Gastric Adenocarcinoma Patients from the Chinese Population
Source: PLoS One. 2015 Apr 17;10(4):e0123985. doi: 10.1371/journal.pone.0123985 (PMC4401731; doi:10.1371/journal.pone.0123985)
Supplement: S3 Table — (PDF) [file pone.0123985.s003.pdf]

**S3 Table:** Area under the curve (AUC) and sensitivity of individual proteins and combinations of proteins between healthy controls and metastatic GA samples (H vs M).

| Protein | AUC (95% CI) | p val | Specificity (%) | | | |
|---|---|---|---|---|---|---|
| | | | 90 | 95 | 99 | 100 |
| OPN | 0.93 (0.91 - 0.95) | 1.14 $x10^{-27}$ | 79.69 | 67.19 | 42.19 | 9.38 |
| sVCAM1 | 0.72 (0.69 - 0.75) | 2.49 $x10^{-8}$ | 39.88 | 29.31 | 15.11 | 10.57 |
| AGP | 0.52 (0.48 - 0.55) | 7.12 $x10^{-1}$ | 6.17 | 4.12 | 2.47 | 1.23 |
| SAA | 0.83 (0.80 - 0.86) | 2.67 $x10^{-17}$ | 64.06 | 54.69 | 29.69 | 18.75 |
| CRP | 0.74 (0.70 - 0.77) | 1.88 $x10^{-9}$ | 50.00 | 39.06 | 28.12 | 10.94 |
| GRO | 0.64 (0.60 - 0.68) | 9.97 $x10^{-4}$ | 34.92 | 34.92 | 6.35 | 1.59 |
| **OPN+sVCAM1+AGP** | **0.95 (0.94 - 0.96)** | **$<10^{-99}$** | **83.13** | **67.61** | **35.22** | **15.97** |
| **OPN+sVCAM1+SAA** | **0.96 (0.96 - 0.97)** | **$<10^{-99}$** | **84.96** | **76.36** | **53.06** | **29.92** |
| **OPN+sVCAM1+CRP** | **0.96 (0.95 - 0.96)** | **$<10^{-99}$** | **85.53** | **72.83** | **44.53** | **35.53** |
| OPN+sVCAM1+GRO | 0.94 (0.94 - 0.95) | $<10^{-99}$ | 77.71 | 66.72 | 39.01 | 27.24 |
| OPN+AGP+SAA | 0.93 (0.92 - 0.94) | $<10^{-99}$ | 77.57 | 70.66 | 42.24 | 1.23 |
| OPN+AGP+CRP | 0.91 (0.90 - 0.92) | $<10^{-99}$ | 78.11 | 67.72 | 37.64 | 9.13 |
| OPN+AGP+GRO | 0.91 (0.90 - 0.92) | $<10^{-99}$ | 70.80 | 60.70 | 47.86 | 1.53 |
| OPN+SAA+CRP | 0.92 (0.91 - 0.92) | $<10^{-99}$ | 73.19 | 64.37 | 46.23 | 26.00 |
| OPN+SAA+GRO | 0.92 (0.91 - 0.93) | $<10^{-99}$ | 76.46 | 65.23 | 48.77 | 32.92 |
| OPN+CRP+GRO | 0.92 (0.91 - 0.92) | $<10^{-99}$ | 72.80 | 70.97 | 51.82 | 34.50 |
| **OPN+sVCAM1+AGP+SAA** | **0.96 (0.96 - 0.97)** | **$<10^{-99}$** | **88.08** | **76.78** | **58.20** | **30.03** |
| OPN+sVCAM1+AGP+CRP | 0.95 (0.94 - 0.96) | $<10^{-99}$ | 81.75 | 72.56 | 43.10 | 33.76 |
| OPN+sVCAM1+AGP+GRO | 0.95 (0.94 - 0.95) | $<10^{-99}$ | 81.88 | 67.89 | 49.28 | 30.68 |
| **OPN+sVCAM1+SAA+CRP** | **0.96 (0.95 - 0.96)** | **$<10^{-99}$** | **83.56** | **74.81** | **50.54** | **27.19** |
| **OPN+sVCAM1+SAA+GRO** | **0.95 (0.94 - 0.96)** | **$<10^{-99}$** | **82.55** | **72.87** | **61.44** | **29.77** |
| OPN+sVCAM1+CRP+GRO | 0.94 (0.94 - 0.95) | $<10^{-99}$ | 78.85 | 73.40 | 56.57 | 35.42 |
| OPN+AGP+SAA+CRP | 0.92 (0.91 - 0.93) | $<10^{-99}$ | 76.31 | 66.92 | 45.69 | 5.38 |
| OPN+AGP+SAA+GRO | 0.91 (0.90 - 0.92) | $<10^{-99}$ | 74.92 | 65.65 | 41.37 | 0.64 |
| OPN+AGP+CRP+GRO | 0.91 (0.90 - 0.92) | $<10^{-99}$ | 75.39 | 67.76 | 38.47 | 0.78 |
| OPN+SAA+CRP+GRO | 0.91 (0.90 - 0.92) | $<10^{-99}$ | 74.92 | 65.31 | 49.84 | 32.74 |
