# Supplementary material for: Inflammatory Serum Proteins Are Severely Altered in Metastatic Gastric Adenocarcinoma Patients from the Chinese Population
Source: PLoS One. 2015 Apr 17;10(4):e0123985. doi: 10.1371/journal.pone.0123985 (PMC4401731; doi:10.1371/journal.pone.0123985)
Supplement: S4 Table — (PDF) [file pone.0123985.s004.pdf]

S4 Table: Area under the curve (AUC) and sensitivity of individual proteins and combinations of proteins between non-metastatic and metastatic samples (NM vs M).

Protein	AUC (95% CI)	p val	Specificity (%)			
			90	95	99	100
OPN	0.74 (0.70 - 0.78)	1.91 $x10^{-7}$	28.12	26.56	18.75	12.50
sVCAM1	0.63 (0.58 - 0.67)	5.48 $x10^{-3}$	26.56	23.44	9.38	7.81
AGP	0.79 (0.75 - 0.82)	4.44 $x10^{-10}$	42.19	34.38	21.88	21.88
SAA	0.77 (0.73 - 0.81)	6.46 $x10^{-9}$	42.19	25.00	18.75	3.12
CRP	0.77 (0.73 - 0.81)	4.09 $x10^{-9}$	45.31	29.69	12.50	12.50
GRO	0.70 (0.66 - 0.74)	1.63 $x10^{-5}$	30.16	14.29	3.17	1.59
**OPN+sVCAM1+AGP**	**0.81 (0.80 - 0.82)**	**$<10^{-99}$**	**52.22**	**41.42**	**17.46**	**6.66**
OPN+sVCAM1+SAA	0.76 (0.75 - 0.77)	1.64 $x10^{-72}$	41.32	25.65	13.82	7.99
OPN+sVCAM1+CRP	0.78 (0.77 - 0.79)	5.44 $x10^{-86}$	49.47	36.35	13.42	10.11
OPN+sVCAM1+GRO	0.75 (0.74 - 0.76)	1.76 $x10^{-64}$	42.45	24.06	11.16	2.99
OPN+AGP+SAA	0.80 (0.79 - 0.82)	8.33 $x10^{-94}$	45.54	35.37	22.69	15.18
**OPN+AGP+CRP**	**0.83 (0.82 - 0.84)**	**$<10^{-99}$**	**53.13**	**38.68**	**17.66**	**4.65**
**OPN+AGP+GRO**	**0.81 (0.80 - 0.82)**	**1.43 $x10^{-97}$**	**46.58**	**32.59**	**14.94**	**0.95**
OPN+SAA+CRP	0.81 (0.80 - 0.82)	$<10^{-99}$	51.21	33.94	18.18	8.94
OPN+SAA+GRO	0.78 (0.77 - 0.79)	4.32 $x10^{-84}$	39.26	29.45	10.74	5.98
OPN+CRP+GRO	0.78 (0.77 - 0.79)	2.36 $x10^{-78}$	41.82	34.57	18.55	5.40
OPN+sVCAM1+AGP+SAA	0.80 (0.78 - 0.81)	5.99 $x10^{-93}$	45.73	37.80	21.19	9.15
**OPN+sVCAM1+AGP+CRP**	**0.81 (0.80 - 0.82)**	**1.95 $x10^{-99}$**	**48.12**	**42.48**	**22.73**	**9.40**
**OPN+sVCAM1+AGP+GRO**	**0.81 (0.80 - 0.82)**	**3.55 $x10^{-98}$**	**52.89**	**41.32**	**14.95**	**4.02**
OPN+sVCAM1+SAA+CRP	0.78 (0.77 - 0.79)	3.08 $x10^{-81}$	49.68	29.27	15.98	9.49
OPN+sVCAM1+SAA+GRO	0.78 (0.77 - 0.80)	6.42 $x10^{-85}$	42.92	31.38	12.92	4.00
OPN+sVCAM1+CRP+GRO	0.76 (0.75 - 0.77)	1.07 $x10^{-69}$	39.22	31.37	12.75	2.94
**OPN+AGP+SAA+CRP**	**0.80 (0.79 - 0.82)**	**1.01 $x10^{-95}$**	**45.30**	**37.29**	**19.26**	**6.63**
OPN+AGP+SAA+GRO	0.80 (0.78 - 0.81)	5.03 $x10^{-90}$	42.77	35.38	20.13	4.25
OPN+AGP+CRP+GRO	0.79 (0.78 - 0.80)	9.96 $x10^{-89}$	48.05	36.97	19.97	1.87
OPN+SAA+CRP+GRO	0.78 (0.76 - 0.79)	2.95 $x10^{-79}$	41.39	30.17	15.17	3.79
